# Influences on Physicians’ Participation in Coordinated Ambulatory Cardiology Care: A Mixed-Methods Study

**DOI:** 10.5334/ijic.5495

**Published:** 2020-11-25

**Authors:** Patrick Hennrich, Regine Bölter, Michel Wensing

**Affiliations:** 1 Heidelberg University Hospital, Department of General Practice and Health Services Research, Im Neuenheimer Feld, Heidelberg, DE

**Keywords:** participation, selective contract, cardiology, coordination, ambulatory care, integrated care

## Abstract

**Introduction:**

In 2009 a managed care programme for coordinated ambulatory cardiology care was established in Southern Germany. Designed as a voluntary contract between health insurers and ambulatory medical specialists, it aims for a guideline-oriented, efficient health care by general practitioners and medical specialists. In this study, we aimed to identify factors associated with physicians’ participation and their relation to the aims of the programme.

**Methods:**

A mixed-methods study was designed. We conducted semi-structured interviews with a sample of 21 specialists participating and 11 specialists not participating in the programme. Structured questionnaires were sent to all eligible medical specialists, of whom 75 specialists participating and 21 specialists not participating in the programme responded. Both the interview and questionnaire covered a range of questions on the participation and implementation of the program.

**Results:**

Financial benefits were the most frequently named reason to participate. Other prevalent motives were the prospect of an alternative to regular health care, expected diagnostic possibilities and recommendations from peers. Reasons for not participating were mainly structural, such as technical modifications as well as economic investments and fear for one’s professional autonomy.

**Conclusion:**

Physicians’ participation in the programme was mainly financially driven and largely unrelated to its care-related aims. Still, it is unclear if these divergences between motivation to participate and aims of a managed care programme affect its eventual impact, hence further research is required.

## Introduction and background

The German healthcare system is highly fragmented, which provides challenges to the coordination of patient care within and between healthcare sectors [1]. Within ambulatory healthcare, several programmes to foster integration have been launched, such as disease management programmes and general practitioner-centred care. More recently, programmes to enhance the quality of ambulatory specialist care and its coordination with general practitioners’ care have been initiated. Legally based on §73c (§140a since 2015) of Book Five of the German Social Security Law (SGB V), these programmes represent so-called “special ambulatory care” [2]. Research evidence on the uptake and impact of these programmes is limited.

The first of these programmes for ambulatory specialist care (“FacharztProgramm”) was launched December 2009 in Baden-Wuerttemberg (a state with approximately 11 million inhabitants) by health insurers AOK Baden-Wuerttemberg and Bosch BKK. The programme is a medical specialists’ complement to general practitioner-centred care and encompasses selective contracts with medical specialists from various fields. Until today, eight medical disciplines have been included: cardiology (since 2009/2010), gastroenterology (since 2011), psychiatry/neurology/psychotherapy (since 2012), orthopaedics/surgery (since 2014), urology (since 2016), diabetology (since 2017), rheumatology (since 2018) and nephrology (since 2020). Participating patients agree to only attend medical specialists who take part in the programme. While participating medical specialists enter a selective contract in their respective field, patients can only participate in the programme as a whole and not in a single medical discipline. Hence, they are subject to the regulations in all of the selective contracts included in the programme at once.

Being the first German selective contract of this kind and the starting point of the medical specialist’s programme in Germany, the contract for ambulatory cardiology practices (“Facharztvertrag Kardiologie”) with the resulting medical specialist’s programme (we will subsequently refer to the whole concept as cardiology programme) had a pioneering role. Key aspects of the programme to enhance and coordinate ambulatory care are: reduced waiting times for enrolled patients regarding appointments (waiting time maximum of two weeks) and in the waiting room on the day of their appointment (waiting time maximum of 30 minutes); the possibility for physicians to offer diagnostic measures and treatments which are normally only remunerated in the inpatient sector to reduce hospitalisations; strict adherence to clinical guidelines by physicians for an evidence-based treatment; preferred prescription of generic medicaments for reasons of cost efficiency; structured communication between medical specialists and general practitioners and vice versa via prefabricated forms; as well as patient-related services such as consultation hours in the evenings [3]. Participation in the programme is financially attractive for medical specialists through increased remunerations compared to regular health care [3]. Shared rules and approaches allow for a horizontal, systemic integration [4] focused on the long-term treatment of chronically ill cardiovascular patients within primary health care.

The programme bears attributes of managed care such as selective contracting, additional payments based on performance combined with lump-sums per service and set standards for the provision of medical care [5,6,7]. It focuses on treating chronic cardiovascular conditions, especially coronary heart disease, heart failure, cardiac arrhythmia and (congenital) heart defects [3]. The number of medical specialists participating in the cardiology programme was high at the time of our study, with 212 physicians in 2018. We were able to identify 99 non-participants through searches in publicly available databases [8], implying estimated participation rate was 68% for 2018, respectively.

As outlined above, the cardiology programme offers additional services to participating patients, aims to standardise communication structures and allows physicians to provide services otherwise only available in the inpatient sector, hence making it an innovative alternative to regular health care in Germany. It can therefore be considered an innovation, which was taken up by a proportion of the targeted population of cardiologists [9]. Its participants outnumber non-participants as data above indicated, although this does not necessarily reflect a comprehensive uptake of all its components. The cardiology programme requires a range of organizational and clinical changes in the ambulatory practices, which has implications for their information technology, administrative procedures, and clinical routines [3]. While participating physicians receive additional payment, money by itself does not necessarily cause these changes [10]. Hence, taking the prerequisites into regard, proper implementation of managed care programmes like the cardiology programme might not come without any difficulty and therefore cannot be taken for granted.

In a previous study, we showed that actual adherence to components of the cardiology programme by physicians was mixed – with standards for medical care itself proved to be well adhered to, enhanced access to cardiology care for patients and information exchange between general practitioners and medical specialists less so [11]. Possible reasons were related to the feasibility of certain requirements in routine practice as well as actual demand for services required in the programme [11].

The physicians’ decision on whether to join the cardiology programme or not was not explored in this previous study. The decision to participate in a managed care programme may be influenced by a variety of factors, which may differ, explicitly or implicitly [12], from its aims to various degrees. For instance, for the cardiology programme a first crude exploration in 2015 showed that financial considerations seemed predominant in the physicians’ decision to participate [13]. At worst, a programme’s intentions are completely contradictory to the motives of the participant. These differences might affect actual implementation by physicians and, in the long run, reduce the impact of a programme. This study therefore aims to explore and understand in-depth what drives physicians to participate or not to participate in a managed care programme like the cardiology programme and how their motivation relates to the programme’s aims. Eventually, this can provide a basis for policymakers when it comes to developing suitable incentive strategies to attract participants in managed care programmes without jeopardising a programme’s implementation and impact on health care.

## Methods

In the context of a larger evaluation study on the cardiology programme (also covering outcomes evaluation, health economics evaluation and a patient survey) conducted by the Goethe-University Frankfurt am Main, aQua-Institute (Institute for Applied Quality Improvement and Research in Health Care) Göttingen and the University Hospitals Jena and Heidelberg, we conducted a mixed-methods process evaluation of the programme. The process evaluation focused on participation in and implementation of the programme by participating physicians as well as on identifying potential impact mechanisms and contextual factors affecting these aspects. To a lesser degree, it also covered the programme’s impact as perceived by physicians. The Medical Research Council Guidance on process evaluations of complex interventions was used as guidance [14]. The study has been conducted in accordance with the Declaration of Helsinki and was approved by the ethics committee of the medical faculty of Heidelberg under reference number S-415/2017. A more detailed description of the study itself and the methodological approach has been published previously [11], the following description is hence slightly condensed.

### Study population and sampling

Eligible were all specialists in internal medicine, who (co-)owned an ambulatory practice in the state of Baden-Wuerttemberg and provided cardiology care. Physicians without a license from the Association of Statutory Health Insurance Physicians (which covers roughly 90% of the population), salaried physicians (neither practice owners nor co-owners) and physicians in hospitals were not included. Participants in the programme were extracted from a list provided by the health insurer, while non-participants were identified through internet databases (for details see [11]).

To ensure diversity of participants and statements, sampling for the qualitative study considered practices as a whole instead of individual physicians within each practice. Therefore, instead of inviting all physicians in a given practice, in every practice eligible for the qualitative study only one registered physician was chosen randomly and invited to participate in the study. Otherwise, a strong overlap of statements and attitudes was to be expected. This resulted in a total of 139 specialists’ practices in the cardiology programme and 84 non-participating practices. All of them were invited. The specialists in the programme received one reminder, the non-participating specialists received two because of the low response rate. All participating physicians provided written informed consent for the telephone interview.

In the quantitative study, contrary to the qualitative approach, instead of one physician, all physicians within each practice were approached, hence the difference in sample sizes. From updated searches and participant lists, we identified a total of 212 medical specialists participating in the cardiology programme and 99 who were not participating. All physicians received a structured questionnaire including an accompanying letter and a post-paid envelope. As the questionnaire was mailed anonymously, completing and returning it was interpreted as consent – a separate declaration of consent was not required here. Contrary to the qualitative study, reminders could not be mailed out directly, as the anonymous approach made it impossible to identify physicians who had not sent back their questionnaire yet. Still, we mailed out one letter to all physicians reminding them of the study in general and asking them to send back their questionnaire if it hadn’t been done already.

### Data collection and measures

The qualitative study was conducted between September 2017 and July 2018. After the appointment for the interview had been made, participants received a short, written questionnaire in preparation of the interview and for reasons of sample description. For medical specialists participating in the cardiology programme it included 30 questions on the physicians, the practice they were based in, patient care and the cardiology programme. For medical specialists not participating in the programme it included 23 questions on the physicians, the practice they were based in and patient care.

Telephone interviews then were conducted supported by a semi-structured interview guide, which was tailored to the respective group of physicians. The interview guide addressed key components of the cardiology programme and aspects that emerged during pilot testing. Six topics were covered: a) reasons for (refraining from) participation in the cardiology programme as well as one’s own expectations, b) day-to-day tasks in patient care and changes one noticed since participation in the programme, c) cooperation with other care providers, especially general practitioners, d) how one organised their practices, e) factors that affected implementation of the programme and f) concomitants of the programme. Pilot testing of the guideline was conducted with 3 physicians. Interviews were planned to last 30 to 40 minutes.

The quantitative, structured survey was conducted between October 2018 and January 2019 through the questionnaires mentioned under “Study population and sampling”. These were constructed by the researchers using multiple choice questions, semantic differential scales, open ended questions and dichotomous questions, spanning four topics: a) general information on each physician and their practice, b) participation in the cardiology programme and its implementation, c) healthcare within one’s practice and d) cooperation with other care providers. Questionnaires were pilot tested with physicians and slightly adapted afterwards.

### Analyses

All telephone Interviews were transcribed verbatim and analysed using MAXQDA 2018. We followed the method of a content-structuring qualitative content analysis as described by Kuckartz [15]. The code system we used was initially deducted from the research question and subsequently inductively complemented by new insights gained during the interviewing process. Texts were manually scanned for important and possibly relevant statements, which were noted as memos. Subsequently, main categories were developed, derived of the research questions, interview guidelines and emerging from topics mentioned by interviewees. All main categories were applied to parts of the empirical material and concerted between researchers to ensure quality and precision in definitions. Concerted main categories were applied to the whole material and further differentiated. Throughout the application, corresponding sub-categories were created inductively on the basis of statements within the interviews. This led to the final code system which was once again concerted between researchers, applied to all transcripts and interpreted subsequently with respect to the main topics of the study and in consideration of frequencies of mentions by the interviewees.

The quantitative data obtained was tested for normal distribution using the Kolmogorov-Smirnov-Test in SPSS 24. The analysis was largely descriptive with a focus on mean values and standard deviations (sd) (median values and corresponding interquartile range (IQR) where data was not normally distributed) as well as frequencies. For mean and median values, the corresponding n is given in square brackets where data was missing. Frequencies are given in absolute numbers with valid percentages respectively.

## Results

In the qualitative study, of the 139 contacted medical specialists participating in the cardiology programme, 23 (16.5%) agreed to participate. A total of 21 (15.1%) were interviewed. The remaining two could repeatedly not be reached for making an appointment. Of the 84 medical specialists eligible but not participating in the cardiology programme, one turned out to be deceased while 11 (13.3%) physicians agreed to participate and were interviewed.

In the quantitative study, of the 212 contacted specialists participating in the cardiology programme, one mailing was returned to sender. Of the remaining 211 specialists, 75 (35.5%) returned the questionnaire. Of the 99 non-participating specialists, 21 (21.2%) returned the questionnaire.

### Sociodemographic characteristics

Table 1 contains sociodemographic characteristics of all participants in the qualitative study.

**Table 1:** Sociodemographic characteristics of medical specialists in the qualitative study.

Variable	Medical specialists (participating) (n = 21)	Medical specialists (non-participating) (n = 11)

Sex (n (%))		
*male*	81.0	81.8
*female*	19.0	18.2
Age (mean (sd))	57 (6.5)	49 (7.2)
Years of professional experience (mean (sd))	28.1 (7.0)	20.0 (7.3)
Practice based since the year… (mean (sd))	2000 (7.7)	2011 (7.3)
Vocational training (n (%))		
*Specialist for internal medicine*	19 (90.5)	5 (45.5)
*…without focus*	3 (14.3)	0 (0.0)
*…with focus on cardiology*	16 (76.2)	5 (45.5)
*Specialist for internal medicine and cardiology*	3 (14.3)	5 (45.5)
*Other*	3 (14.3)	2 (18.2)
Practice location (n (%))		
*City core*	11 (55.0)	7 (70.0)
*Urban hinterland (~20 km)*	4 (20.0)	2 (20.0)
*Rural area*	5 (25.0)	1 (10.0)
Type of practice (n (%))		
*Individual practice*	6 (30.0)	7 (70.0)
*Shared practice*	2 (10.0)	1 (10.0)
*Group practice*	11 (55.0)	2 (20.0)
*Ambulatory health care centre*	1 (5.0)	0 (0.0)
Individual patients per quarter (n (%))		
*<500*	0 (0.0)	1 (9.1)
*500–1000*	7 (33.3)	5 (45.5)
*1001–1500*	6 (28.6)	4 (36.4)
*>1500*	8 (38.1)	1 (9.1)
Full-time positions (physicians) (mean (sd))	2.9 (3.4)	1.7 (1.0)
Full-time positions (physician’s assistants) (mean (sd))	6.2 (6.1)	3.7 (2.6)
Percentage of AOK-patients participating in the medical specialist’s programme (mean (sd))	43.9 (19.3)	-
Physician’s participation in the cardiology programme since the year… (mean (sd))	2011 (1.2)	-

Table 2 contains sociodemographic characteristics of all participants in the quantitative study.

**Table 2:** Sociodemographic characteristics of medical specialists in the quantitative study.

Variable	Medical specialists (participating) (n = 75)	Medical specialists (non-participating) (n = 21)

Sex (n (%))		
*male*	60 (80.0)	18 (85.7)
*female*	15 (20.0)	3 (14.3)
Age (median (IQR))	56 (51–60)	54 (45–57)
Years of professional experience (median (IQR))	28.0 (23.0–32.0)	23.0 (18.5–29.7)
Practice based since the year… (mean (sd))	2003 (8.2)	2009 (8.5)
Vocational training (n (%))		
*Specialist for internal medicine*	62 (82.7)	16 (76.2)
*…without focus*	9 (13.0)	1 (4.8)
*…with focus on cardiology*	47 (68.1)	15 (71.4)
*Specialist for internal medicine and cardiology*	22 (29.3)	6 (28.6)
*Other*	6 (8.0)	2 (9.5)
Practice location (n (%))		
*City core*	53 (72.6)	12 (57.1)
*Urban hinterland (~20 km)*	8 (11.0)	6 (28.6)
*Rural area*	12 (16.4)	3 (14.3)
Type of practice (n (%))		
*Individual practice*	17 (23.6)	7 (33.3)
*Shared practice*	14 (19.4)	2 (9.5)
*Group practice*	37 (51.4)	10 (47.6)
*Ambulatory health care centre*	4 (5.6)	2 (9.5)
Individual patients per quarter (n (%))		
*<500*	2 (2.7)	3 (14.3)
*500–1000*	34 (45.9)	10 (47.6)
*1001–1500*	21 (28.4)	3 (14.3)
*>1500*	17 (23.0)	5 (23.8)
Number of full-time positions (physicians) (mean (sd)) [n]	3.0 (2.7) [66]	2.3 (2.1) [20]
Full-time positions (physician’s assistants) (mean (sd))		
*0 up to 3*	17 (22.7)	4 (20.0)
*More than 3, up to 6*	27 (36.0)	10 (50.0)
*More than 6, up to 10*	18 (24.0)	3 (15.0)
*More than 10*	13 (17.3)	3 (15.0)
Percentage of AOK-patients participating in the medical specialist’s programme (mean (sd))	19.4 (11.8)	-
Physician’s participation in the cardiology programme since the year… (mean (sd))	2012 (2.6) [60]	-

### Motivation to participate in the cardiology programme

Results of the quantitative study regarding physicians’ motivations to participate in the cardiology programme are shown in Table 3. Physicians were asked to tick a maximum of 3 reasons why they decided to participate.

**Table 3:** Reasons to participate in the cardiology programme as mentioned by medical specialists.

Participation in the cardiology programme because of… (n (%))	Medical specialists (participating) (n = 75)

…receiving higher reimbursement than in regular health care	60 (80.0)
…it being an alternative to the statutory health insurance system	50 (66.7)
…it providing more diagnostic possibilities than in regular health care	25 (33.3)
…easier accounting than in regular health care	24 (32.0)
…a recommendation by the professional association	17 (22.7)
…closer cooperation with general practitioners than in regular health care	13 (17.3)
…having more time for patients than in regular health care	10 (13.3)
…a higher guideline-orientation than in regular health care	8 (10.7)
…participation of or a recommendation from colleagues	6 (8.0)
Other	8 (10.7)

In the qualitative study, we identified a total of 7 categories with 14 sub-categories in the statements on motivation to participate in the programme. A complete overview on all (sub-)categories can be found in Table 4. For each (sub-)category we included one representative quote, the physicians’ names were replaced by an identification number. Other names, places and potentially unique identifiers were substituted by a place holder in upper case letters put in square brackets. Our additions for a better understandability of several statements were placed in lower case letters and put in square brackets as well. All quotations were translated by us from German to English carefully. Wherever a literal translation proved to be ambiguous, we rephrased it with the aim of conveying its original meaning.

**Table 4:** Overview of categories and subcategories of incentives to participate in the cardiology programme.

Category	Sub-category	Number of statements on the category (Number of physicians with statements)

**External incentives** (category had been asked for explicitly)		**29 (21)**
	Colleagues’ attitudes towards the cardiology programme (see section “Motivation through peers”)	27 (21)
	Advertising/peer pressure (from other physicians) ID-2: “[…] And as a physicians’ organization…and the [ORGANIZATION] was very strong in this area, they…very early they raised solidarity among physicians to some degree.”	2 (2)
**Economic incentives**		**27 (16)**
	Aspects of remuneration (see section “Economic incentives”)	20 (16)
	No cap on patient numbers in the programme ID-10: “[…] And thirdly we’re able to see more patients overall [in the cardiology programme] because in the system of the Association of Statutory Health Insurance Physicians we are budgeted regarding patient numbers.”	5 (4)
	Aspects of accounting ID-4: “Yes, of course, there was dissatisfaction with accounting in the system of the Association of Statutory Health Insurance Physicians, regarding caps on numbers of cases. So, a lot of things that made you dissatisfied beforehand seemed to be better from the outset and this proved to be true for me, yes.”	2 (2)
**Incentives related to reputation** (category had been asked for explicitly)	(see section “Reputational benefits”)	**20 (20)**
**Incentives related to health care**		**19 (11)**
	Better/more services for patients through the programme (see section “Expected improvements related to health care”)	16 (9)
	More medical guideline-oriented care than regular health care ID-8: “Yeah, sure, you might adhere more strictly to guideline-oriented, rational medicine now, yes.”	2 (2)
	Better cooperation with general practitioners than in regular health care ID-9: “[…] So this means, background, maybe better cooperation between general practitioner, medical specialist, a more distinct task sharing.”	1 (1)
**Professional political incentives** (category had been asked for explicitly)		**10 (6)**
	ID-2: “[…] And secondly I’ve been a long-time member of [ORGANIZATION] for political considerations. So, the whole thing was a logical consequence.”	
	ID-13: “[…] so I remember that beforehand these scenarios of leaving the system of the Association of Statutory Health Insurance Physicians had been discussed […]. […] And so that these, let’s call it politicisations of this dispute, were already advanced. And so one of these reasons for participating in this alternative system [the cardiology programme] was definitely also a political one. […]”	
**Structural incentives**		**8 (5)**
	Binding of patients ID-5: “[…] I did not want to give off patients to other colleagues, say if someone participates in the medical specialist’s programme, especially after orthopaedics and gastroenterology started, it was important to me that I could still take care of the patients I already had.”	2 (2)
	Alternative to/advancement compared to the system of the Association of Statutory Health Insurance Physicians ID-5: “Being a health economist I know that something has to change in the system of statutory health insurance physicians or in the overall health care system, that we need a paradigm shift within the health care system, that we can’t manage this through a total upheaval but need sub-steps and I classify the system of selective contracts as a small or maybe even a big step in this change in system. […]”	2 (2)
	High percentage of patients insured through AOK/Bosch BKK ID-8: “[…] sure, in the beginning only AOK was involved. […] Sure, my clientele here contains a relatively high percentage of AOK-patients, right? Sure, if you only have two percent of AOK-patients you need to think about what you’re going to do. […]”	1 (1)
	Referral within the programme is only possible between participating physicians ID-11: “[…] The situation is that only medical specialists who participate may be chosen or referred to. This was the original idea. That’s why it made sense to participate in it of course. So that general practitioners are able to refer to a medical specialist who also participates in the programme.”	1 (1)
	Taking over an already participating practice ID-12: “My predecessor was one of the first participants in the programme. I joined later and started to participate in the programme as well. So I continued with an existing system. […]”	1 (1)
	Hope for a successful implementation of software ID-8: “[…] partially, it was no insignificant effort software-wise. I had, well, since I’m practice-based I had relatively great faith in my software-provider to wangle it properly. Other colleagues had a lot more difficulties I think.”	1 (1)
**Incentives related to personal background/involvement in the underlying contract**		**4 (3)**
	ID-4: “So, of course I know [PERSON] pretty well, who was involved in negotiating the contract […]. So I witnessed a lot of things there and that influenced me of course. […] So for us it was clear from the get-go because we were very close to the origination [of the cardiology programme] and I noticed how they negotiated and so on. Just because I knew the participants in the negotiations [personally], so it was clear for us to participate from the get-go. […]”	
	ID-9: “I am a member of [ORGANIZATION] and tracked the development of the contracts and also got to know the general framework during the development phase. This clearly made me decide for this kind of contract.” ID-22: “The main reason was that I’m a board member of [ORGANIZATION] and therefore was already involved in the development of the contract. […] And therefore it was clear for me to participate in the programme myself.”	
	ID-22: “The main reason was that I’m a board member of [ORGANIZATION] and therefore was already involved in the development of the contract. […] And therefore it was clear for me to participate in the programme myself.”	

In the subsequent sections, the results of the qualitative and quantitative studies are presented thematically.

### Economic incentives

The vast majority mentioned higher reimbursement as compared to regular health care as a motivation to participate in the programme. This is supported by insight gained from the qualitative interviews. These showed that more than 75% of participating physicians deemed economic incentives important, especially financial benefits in terms of higher remuneration compared to regular health care and the discontinuation of caps on patient numbers. Physicians sporadically felt not only remunerated higher, but also perceived more (financial) recognition of their performance.

ID-3: “Because I saw a positive effect for me, regarding patient care and financially as well, of course, because it’s more attractive than the lump-sums I get from the Association of Statutory Health Insurance Physicians and I have more possibilities to check the patient, for example, and so on, and yes. And some things are remunerated, respectively remunerated at all, and remunerated better.”

A further economic aspect was the expectation that accounting would be easier than in regular health care, mentioned by 32.0% of physicians. In the qualitative study, however, this was only mentioned in isolated cases.

### Structural incentives

Especially the quantitative study showed that structural and systemic reasons played a role for medical specialists. 66.7% stated that one of their reasons for participation in the cardiology programme was because it is an alternative to regular health care in Germany as it is offered by statutory health insurance for patients who do not participate in managed care programmes or similar types of care. In the qualitative study, isolated physicians brought this topic up as well and perceived the cardiology programme as a paradigm shift away from the current health care system or as being more transparent regarding accounting aspects. An additional structural aspect mentioned in the interviews was, among others, the wish to bind or keep patients who wanted to participate in the programme and were cared for by the practice in the past.

### Expected improvements related to health care

Several health care-related expectations were deemed relevant for participation in the cardiology programme by medical specialists in the quantitative study. Especially the diagnostic possibilities available to participants in the programme were named in this regard. Cooperation with general practitioners, time available for individual patients and guideline-orientation of care played a further, yet less prevalent role.

In the qualitative study, reported incentives related to health care were mainly an (expected) optimization of patient-related services through the programme. The physicians justified this expectation with more diagnostic possibilities, a closer monitoring of the patients than in regular health care and higher availability of appointments. Diagnostically the possibility to offer services normally performed by hospitals in an ambulatory setting was highlighted. Such changes in health care within the programme were often derived from the increased remunerations for participating physicians, in the sense of an incentive or possibility to offer more encompassing services because of a higher remuneration.

ID-7: “[…] Furthermore the programme contains services we can only offer there and not for patients insured in the collective contract – be it ambulatory cardioversion, implantation of heart defibrillators, ICD box changes, these are all services suited to be offered ambulatory but normally not remunerated and they were included in the programme and this makes the programme interesting of course.”

### Motivation through peers

Motivation to participate because of others’ recommendations played a role as well. The quantitative study showed that this was especially the case when it came to recommendations by the professional association the specialists were members of. Recommendations from colleagues were mentioned more seldom.

In the qualitative study, a more substantial part of physicians named the positive or encouraging attitude of colleagues towards the cardiology programme or their participation as an external incentive to participate themselves. Those who did not report direct influence of their colleagues still reported a majoritarian positive attitude towards the programme in their local medical setting. About a third of physicians perceived their colleagues’ attitudes towards the programme as highly diverse.

ID-1: “[…] And somebody should do it in [PLACE], it’s no good if all of us just wave it aside and see it as too much of an effort and yes, these kind of were the reasons and Mister [NAME] in [PLACE] actually also…motivated me to do it, so I thought: Ok, I’ll try it.”

Researcher: “So this means the opinion of colleagues was decisive for participation.”

ID-1: “Yes.”

### Reputational benefits

Expectations related to an influence of participation in the programme on the physicians’ reputation were explicitly surveyed in the qualitative study (hence the high number of mentions) but seldom found – more than 50% of physicians reported that considerations on their own reputation had no effects whatsoever on their decision to participate in the programme.

ID-6: “Reputation is a big word. I would rather say no. […] No, if I had to, with one word, no. It’s definitely true that patients appreciate it [the medical specialist’s programme] but if you would want to call it reputation, it’s rather a positive attribute of a practice, I’d rather not call it reputation.”

## Inhibiting factors for participation in the cardiology programme

Results of the quantitative study on physician’s motivations not to participate in the cardiology programme are shown in Table 5. Physicians were asked to tick a maximum of 3 reasons why they decided not to participate.

**Table 5:** Reasons not to participate in the cardiology programme as mentioned by medical specialists.

No participation in the cardiology programme because of… (n (%))	Medical specialists (not participating) (n = 21)

…administrative efforts	14 (66.7)
…costs	6 (28.6)
…necessary modification of information technology	6 (28.6)
…inability or reluctance to fulfil all of the contractual terms	6 (28.6)
…fear for the survival of the statutory health insurance system	4 (19.0)
…fear for one’s professional autonomy	3 (14.3)
…professional political aspects	2 (9.5)
…a regional lack of general practitioners in general practitioner-centred care	2 (9.5)
…colleagues advised against participation	2 (9.5)
…not knowing about the cardiology contract	2 (9.5)
…a lack of suitable patients	1 (4.8)
Other	5 (23.8)

For the qualitative statements of the 11 physicians not participating in the cardiology programme on inhibiting factors we identified 6 categories with 22 sub-categories. A complete list of (sub-)categories can be found in Table 6.

**Table 6:** Overview of categories and subcategories of inhibiting factors of participation in the cardiology programme.

Category	Sub-category	Number of statements on the category (Number of physicians with statements)

**Structural inhibiting factors**		**25 (8)**
	Implementation efforts (see section “Structural inhibiting factors”)	14 (7)
	New practice ID-15: “I entered the practice as recently as 2014, I entered the structures, the structures did not allow for it [the cardiology programme]. […]”	4 (4)
	Computer issues ID-14: “Furthermore I need a more powerful computer if I’m unlucky because the current one will become too slow then for the VPN, so I don’t personally see this financial benefit for me being that exorbitant I have to say.”	4 (3)
	Local lack of participating patients ID-16: “[…] economically it did not pay off, there were too few patients participating. […]”	2 (1)
	High percentage of private patients in practice ID-17: “So here in the practice there is, you have to say, a high percentage of private patients – right, that for sure is a reason why you say: ‘Well, okay, it [the cardiology programme] is not that vital’.”	1 (1)
**External inhibiting factors** (category had been asked for explicitly)		**15 (11)**
	Colleagues’ attitudes towards the cardiology programme (see section “External inhibiting factors”)	12 (11)
	Negative experiences of colleagues ID-17: “Yes, indeed I adopted a practice. […] And with it I kind of adopted the status too, as it was conveyed to me that it [the cardiology programme] wasn’t necessarily favourable.”	3 (2)
**Other inhibiting factors**		**15 (5)**
	The programme stimulates an intentionally wrong coding of cases (see section “Other inhibiting factors”)	3 (2)
	Individual principles (see section “Other inhibiting factors”)	2 (2)
	No benefits for participating patients ID-20: “Well, the physicians receive more money, but I think that…well, I imagine that independent of the programme I don’t take worse care of non-participants than of those in the programme.”	2 (1)
	No examinations at the hospital allowed for participating physicians ID-19: “[…] for a long time, the main reason was that we, our practice, also conducted examinations with an intracardiac catheter but not in a practice but inside the hospital. And…or in the hospital here in [PLACE] and then it wasn’t possible any longer with it [the cardiology programme] back then, merely for practical reasons because it was a requirement of the programme that you, well ‘ambulatory instead of inpatient’, right? […]”	2 (1)
	No benefits of the programme besides remuneration ID-20: “I think it…physicians receive more money, that’s right, but I…I can’t…I don’t see any improvement.”	1 (1)
	Appointments within the programme are not prioritised by urgency ID-20: “And they come here urgently and say: ‘We need to wait for half a year’, I can’t understand it. […] this, I think, is sometimes related to the medical specialist’s programme. […] they are not insured by [health insurers offering the programme]. So, they automatically fall through the cracks which I think is highly problematic.”	1 (1)
	Programme worsens physician-patient-relationship ID-18: “I think that health insurers and physicians are two separate institutions who need to fulfil their own tasks each and I think that the physician is, through these health care structures like the medical specialist’s programme, influenced sooner or later regarding autonomy so that the physician-patient-relationship is worsened.”	1 (1)
**Autonomy-related inhibiting factors**		**13 (4)**
	The programme jeopardises professional autonomy (see section “Autonomy-related inhibiting factors”)	6 (2)
	The programme restricts prescriptions (see section “Autonomy-related inhibiting factors”)	2 (2)
	The patient is bereft of their freedom of choice in physicians/therapies (see section “Autonomy-related inhibiting factors”)	2 (2)
	Supervision through health insurers ID-18: “[…] you should rather allow physicians to further prescribe what they deem adequate, if they suitably continue their education, yeah. And not perform any benchmark tests or various checks by the [HEALTH INSURER] because one admits too many patients to the hospital or too few patients. Or if one’s prescribing too much drugs for heart failure or too expensive ones or too cheap ones. […]”	2 (1)
	The programme contains lots of requirements ID-14: “[…] what bothered me as well, I have to say, is that this programme dictates to you a lot. […]”	1(1)
**Economic inhibiting factors**		**3 (3)**
	Disproportion of costs and earnings ID-16: “Yes, so I had to pay higher software license fees, I had to pay rent for the connecting device and what…and my assistant or we had to make a second accounting and in total everything created more work than it was good for financially.”	2 (2)
	Financial barriers Researcher: “[…] simply the investments you have to make…” ID-21: “Correct.”	1 (1)
**Professional political inhibiting factors**		**2 (1)**
	Disempowerment of the Associations of Statutory Health Insurance Physicians (see section “Professional politicial inhibiting factors”)	2 (1)

## Structural inhibiting factors

The vast majority of physicians in the quantitative and qualitative studies reported structural aspects as reasons for not participating in the cardiology programme. Especially additional administrative, technical and personal efforts to implement the whole programme were deemed decisive, furthermore financial and staff resources. The quantitative results added regional structures like the number of eligible patients and general practitioners in general practitioner-centred care.

ID-14: “[…] decided against it [the cardiology programme] up to now just because I can’t go to the additional effort software-wise regarding linkage to the health insurer as well as staff efforts, so that my assistants don’t only know codes for regular accounting but also those required because of the selective contract. I just don’t have enough adequately qualified staff, I have to put it this way.”

## Economic inhibiting factors

The quantitative study showed that financial calculations and cost considerations played a role for nearly one third of physicians in their decision not to participate in the programme. The qualitative study backed this up in sporadic cases, but economic aspects were not mentioned as often during the telephone interviews.

## Professional political inhibiting factors

In the quantitative study, professional political aspects played a role for several physicians. Specifically, a fear for the continued existence of the statutory health insurance’s system was mentioned, accompanied by more general political motivations not to participate in the cardiology programme. Qualitative data showed a singular mention of fear for the continuance of the statutory health insurance’s system as well. Here, the cardiology programme was seen as a threat to regular health care as it transfers regulatory aspects of care from the Associations of Statutory Health Insurance Physicians to the respective health insurance company.

ID-18: “[…] I think it’s a mistake that the Associations of Statutory Health Insurance Physicians are disempowered [by the cardiology programme] and the health insurers gain more and more insight into patients’ conditions as well as more influence on treatment. I think this weakens the Associations of Statutory Health Insurance Physicians […].”

## Autonomy-related inhibiting factors

More than 14% of physicians in the quantitative study and over one third of physicians in the qualitative study chose not to participate in the cardiology programme because of concerns related to freedom – especially out of fear for their own professional autonomy as such or in relation to prescription regulations as well as the patients’ free choice of medical practitioners.

ID-18: “I think professional autonomy is heavily threatened here by the cardiology programme, gastroenterology programme. This tearing of the healthcare landscape to singular contracts will not serve patients or physicians, yes, but only health insurers. Because every health insurer is cherry-picking and going to try to maximise profits through this thing. And this is, I think, a general fallacy of our healthcare system.”

ID-19: “[…] you also get certain regulations regarding the prescription of medicine. […] That’s another thing where you currently still have complete freedom…well, complete, that’s not right either, but more freedom [than within the cardiology programme] […].”

ID-19: “[…] they [patients] are not really pointed to the fact that they’re partially…that they partially abandon their freedom of choice regarding therapy in the ambulatory sector. […]”

## External inhibiting factors

Local colleagues’ attitudes towards the cardiology programme were reported as being mixed or difficult to assess in the qualitative study, only isolated cases reported a clearly positive attitude. None of the physicians interviewed perceived an explicitly negative attitude. The influence of colleagues’ attitudes on the decision not to participate was left unclear in more than 50% of the cases. The quantitative study showed that colleagues’ attitudes were deemed important by roughly 10% of physicians in the study.

ID-18: “About half of them are participating, half of them aren’t. And let me put it this way, the idealists, to which I belong, did not participate. And those who are probably affected more by economic necessities and think they have to earn a little extra money through this medical specialist’s programme, they rather participate.”

Researcher: “And do you think that your own decision not to participate has been somehow influenced by the mood of the colleagues, or…”

ID-18: “No, no.”

## Other inhibiting factors

A lot of inhibiting factors proved to be very heterogeneous, as the category “Other factors” shows quantitatively and qualitatively. For non-participants several specific conditions or regulations of the programme were decisive as well as individual principles and beliefs.

ID-19: “So smaller reasons that always irritated me about the programme is that it offers incentives to make patients sicker than they are, that is to code diagnoses generously which could be done formally but is clinically unnecessary but sticks to the patient, makes him, the patient more ill to be able to bill better [more expensive] modules – that’s what detested me a bit […]”

ID-18: “[…] That’s why, as a matter of principle I do not participate, yes, as I want to show clearly that I want to stay autonomous. I don’t want to be influenced by the [HEALTH INSURER] and be baited by some extra payments. I’m staying autonomous. That’s why I’m a self-employed physician.”

## Discussion

The cardiology programme was designed to enhance access to and quality of ambulatory cardiology care by specifying a range of requirements in a contract and offering additional payment for participation. The primary aims of the cardiology programme such as an improvement of health care played a rather modest role in physicians’ decisions to participate: Especially the prospect of an increased remuneration, structural expectations, the expectation of a broader scope of medical services and a positive attitude of local colleagues were central incentives for physicians to participate in the cardiology programme. Inhibiting factors which were often mentioned were of an administrative, structural or economic kind and accrued partly from very specific, individual considerations or were related to the professional autonomy of the physician.

### Financial motives of physicians and aims of the programme

Even if there seems to be a divergency between the aims of the contract and physicians’ mainly financial motives to participate, this does not automatically imply insufficient implementation or that the programme’s goals cannot be attained: The strong focus physicians put on financial aspects can indicate a function of remuneration as a motivator to optimise one’s own patient care, as international research on incentives for an improvement of medical care quality [16] and integration of care already showed [17], and as the category “Principle: Better reimbursement for better quality” in the short survey mentioned in the introduction indicated [13]. On the other hand, financial incentives need to fulfil several criteria to achieve such effects [18]. Participating physicians named mainly and sometimes solely a higher remuneration as an incentive to participate. Many deemed the quality of their care to be reasonably good and not really influenced by the programme. These perceptions seem to reflect a lack of awareness of the widely prevalent coordination problems in German healthcare from the perspectives of patients and general practitioners [19]. They might lead to participants aiming solely for improvement of their financial situation without changing their own patient care or, in a worst-case scenario, subordinating patient care to financial intentions – a known risk for remuneration schemes in managed care [20]. It has been shown that physicians who receive higher, visit-related remuneration tend to increase the number of patients they see while the time for patients and quality of care decreases [21]. Both are aspects that would be directly opposed to the cardiology programme’s aims.

This situation also points to a possible difference in worlds of thinking: On the one hand there were the developers of the cardiology programme, which aimed at an improvement of quality, efficiency and provision of ambulatory cardiology care. On the other hand, there were the physicians participating in the programme. While the developers of the programme likely saw financial incentives through reimbursement as a necessary mean to attract participants, a good deal of physicians solely focused on this aspect. The developers’ ultimate aim was to improve medical care, the physicians seemed to place the programme largely outside the medical domain instead. A possible reason for this might be the approach of the cardiology programme itself: In recent years, integrated care became increasingly patient-centred with rather holistic approaches, including patients, nurses, social services and other non-medical staff [22,23,24,25]. The cardiology programme however, even though it aims at an optimisation and integration of care in the ambulatory sector, mainly focuses on structuring and enhancing care provided solely by the medical specialist through higher remunerations. Patients and non-medical staff are barely included in this optimisation process. Just like regular health care in Germany, the cardiology programme seems to follow a rather physician-centred approach: Besides their communication with general practitioners, medical specialists in the programme still pretty much work on their own in a more or less unidirectional way towards the patient and do not necessarily need to change their medical daily routine significantly. Hence, from the get-go, the programme might not have been striking to physicians for its medical components, since they were already used to physician-centred care and may rather have appraised the monetary aspects. Here, a broader approach of the cardiology programme, exceeding a purely physician-centred care by including further health care providers and patients, might be a solution to foster integration of care and conquer the risk of participation for purely financial reasons, as the other providers involved would recognise and most likely sanction such behaviour. However, one needs to consider that the German health care system is typically driven by detailed medical and administrative requirements and financial aspects play an important role. Therefore, comprehensive implementation of such an approach seems unlikely at least in the short run. As it has been argued before, this would additionally most likely require a change in physicians’ professional culture beforehand [22].

Still, it remains unclear whether a financially driven participation is in fact problematic here: several physicians in the study explicitly perceived increased remuneration in the cardiology programme as an opportunity to specifically extend and improve their own scope of ambulatory care. A simple claim of financial benefits without resulting changes in patient care is hence not to be expected then. Here, higher remuneration may have influenced patient care positively. Financial incentives were able to make the cardiology programme appealing to some physicians and, at the same time, provided a basis for more regular and tight-knit services, therefore served the purpose of the programme in these cases.

### Influence of colleagues

Physicians participating in the cardiology programme reported an influence of their local colleagues’ attitudes on the decision to participate. When it comes to the diffusion and acceptance of innovations, such interrelations between medical practitioners have already been shown in the 1950s works of Coleman et al. [26,27]. On the other hand, our data did not indicate similar processes for physicians not participating in the programme. This might be a consequence of the differences we observed: Participants perceived rather positive local attitudes towards the programme, while non-participants perceived rather mixed attitudes. The mainly positive attitude in the participants’ environments could have functioned as an incentive for participation. For the non-participants, this aspect might have been absent because of attitudes difficult to assess and therefore suppressing such an external motivation. Another possibility is that the interviewed non-participants might have been less integrated into (informal) social networks with colleagues, which could inhibit such effects when it comes to acceptance of innovations [28] (see also [26]).

### Fear for loss of professional autonomy

The statements of several non-participants showed that there were physicians who have been critical of the contractual binding to the health insurer within the cardiology programme. They especially perceived an emergence of unilateral dependence instead of cooperation. This is likely based on the balance of power within the programme: Health insurer and management organisation have a unilateral controlling function over physicians’ implementation of the programme. Meanwhile, the negotiation power of the individual physician is limited, also because the contract is arranged independent of the Associations of Statutory Health Insurance Physicians. Physicians only have indirect influence through the state-wide association of practice-based cardiologists. For some physicians, this constellation led to a fear of misuse of the contractual relationship in terms of interference in their own professional autonomy. Similar aspects have already been looked into in past research [29] and are comprehensible insofar as the cardiology programme involves a lot of requirements and regulations for participating physicians. It is important to ensure a beneficial behaviour of contractual partners and keep up mutual trust. Certain mandatory regulations, however, are unavoidable to coordinate care and set standards for participants – otherwise, there can’t be a basis from which health care could be optimized as intended by the programme.

### Limitations

Limitations of our study result from the evaluation project itself: Data collection has been conducted between 2017 and 2019 – for some physicians this was eight years after they entered the cardiology programme. After such a time period it is unclear to what extent there are still memories left from the start of participation and preceding considerations. Unbiased and complete reflection on these aspects is especially questionable when it comes to non-participants who possibly never reconsidered their decision not to join and therefore probably did not pay any further attention to the cardiology programme or medical specialist’s programmes in general.

The number of participants in the quantitative study was substantially higher than in the qualitative study. This came as no surprise, as we assumed that filling out the written questionnaire is less time-consuming than completing an interview via phone. Furthermore, the quantitative study involved less personal information as it provided anonymity to the participants, while the qualitative study used pseudonyms and required written, informed consent as well as appointments for the interviews upfront.

The total response rate in the qualitative study was sufficient to reach a saturation of statements in the group of medical specialists participating in the cardiology programme – during the course of the interviews, certain aspects were repeatedly mentioned by various physicians and no additional topics requiring further interviews were brought up. With 35.5%, the quantitative response rate for specialists in the programme was not overly high, but within the scope of response rates studies with physicians typically achieve – especially considering that all medical specialists in the cardiology programme were approached instead of a sample, so the results cover over one third of the whole population. The response rate in the group of non-participating specialists was lower than the rate of participating specialists for both studies. While in the qualitative study we assumed a saturation of arguments in the group of non-participating specialists as well given that after several interviews no new aspects were mentioned, the response rate of 21,2% in the quantitative study left room for improvement and limited further quantitative comparisons between participants in the programme and non-participants.

Methodologically, the mixed methods approach can be seen as advantageous compared to using a single method. The qualitative data partly served as foundation of the quantitative study. Vice versa, it provided deeper insight into quantitative data in terms of possible explanations of quantitative results. The decision to use telephone interviews in the qualitative study was mainly driven by the fact that physicians normally only have little time and personal interviews had required longer appointments, possibly further reducing participation in the study. Still, personal interviews might have led to different results, especially when it came to rather sensitive topics.

The sample composition posed another challenge: It may be possible that physicians with strong opinions were more likely to participate – be it extremely in favour of the programme or extremely hostile towards it. Participants in the study furthermore might have answered socially desirable – regardless of our assurance of confidentiality and protection of the physicians’ identities it is possible that especially critical considerations were reported understated.

## Summary and conclusion

Using the example of the cardiology programme in the German state of Baden-Württemberg, our study explored and analysed what motivates medical specialists in ambulatory cardiology care to take part in a managed care programme. This provides additional knowledge on how to potentially increase participation in managed and integrated care programmes and hence may enable policymakers to spread acceptance of such programmes by tailoring incentives to the needs and expectations of physicians.

Our results showed that financial incentives and administrative issues played an important role for the motivation of physicians to participate or not to participate in the cardiology programme. Increased reimbursement was perceived as an additional source of income and, for several physicians, as a basis to extend their scope of ambulatory care. Depending on which aspect is deemed more important by the individual physician, this can either facilitate or contradict the aims of the programme. Here, more in-depth research seems adequate to further investigate possible effects of personal motives to participate on the actual implementation and eventual impact of such managed care programmes. The importance of colleagues’ positive attitudes for several specialists showed that open-mindedness of local peers towards a managed care programme seemingly enhances the acquisition of physicians as well. At the same time, the limits of acquisition became visible: When they deemed the programme’s administration complex, estimated necessary financial efforts as high or had concerns about their professional autonomy, physicians were reluctant to participate. Furthermore, several medical specialists were unable or unwilling to participate because they were in individually special situations which a broadly designed programme like the cardiology programme was not able to cover.

From what our study showed, policymakers should especially focus on financial attractiveness of a managed care programme to attract a high number of potential participants among physicians. On the other hand, they need to be cautious as it is still not clear if a purely financially motivated participation might limit actual implementation. Further aspects to enhance a programme’s attractiveness proved to be little additional effort for participants when it comes to implementation and a supporting attitude from local peers. At the same time, unavoidable interdependencies between physicians, health insurers and management corporations resulting from the contracts underlying such a programme need to be designed in the light of mutual trust between all parties involved and ideally reduce the risk of imbalances in power, as these might cause doubts among physicians about their professional autonomy. However, even with a considerately designed programme, individual needs and expectations of physicians might still prevent their participation in the end. Here, a more thorough integration of physicians in the design process of managed care programmes could further reduce barriers.
